# Antioxidant and Anti-inflammatory Effects of α-Lipoic Acid on Lipopolysaccharide-induced Oxidative Stress in Rat Kidney

**DOI:** 10.1007/s00005-023-00682-z

**Published:** 2023-06-28

**Authors:** Beata Skibska, Ewa Kochan, Andrzej Stanczak, Anna Lipert, Agnieszka Skibska

**Affiliations:** 1 Department of Applied Pharmacy, Faculty of Pharmacy, Medical University of Lodz, Lodz, Poland; 2 Department of Pharmaceutical Biotechnology, Medical University of Lodz, Lodz, Poland; 3 Department of Sports Medicine, Medical University of Lodz, Lodz, Poland; 4 Department of Biomolecular Chemistry, Medical University of Lodz, Lodz, Poland

**Keywords:** Alpha-lipoic acid, Kidney injury, Oxidative stress, Lipopolysaccharide

## Abstract

α-Lipoic acid (α-LA) is a naturally occurring organosulfur component. Oxidative stress plays an essential role in the pathogenesis of various diseases, such as kidney and cardiovascular diseases, diabetes, neurodegenerative disorders, cancer and aging. Kidneys are especially vulnerable to oxidative stress and damage. The aim of the study was to evaluate the effect of α-LA on lipopolysaccharide (LPS)-induced oxidative stress parameters in rat kidneys. The experimental rats were divided into four groups: I—control (0.9% NaCl i.v.); II—α-LA (60 mg/kg b.w. i.v.); III—LPS (30 mg/kg b.w. i.v.); and IV—LPS + LA (30 mg/kg b.w. i.v. and 60 mg/kg b.w. i.v., respectively). In kidney homogenates the concentration of thiobarbituric acid reactive substances (TBARS), hydrogen peroxide (H_2_O_2_), sulfhydryl groups (−SH), total protein, superoxide dismutase (SOD), total glutathione (tGSH), reduced glutathione (GSH), glutathione disulphide (GSSG) and the GSH/GSSG ratio were determined. In addition, the levels of tumour necrosis factor (TNF)-α, and interleukin (IL)-6 were measured to assess inflammation and was estimated kidney oedema. Studies have shown that α-LA administered after LPS administration attenuated kidney oedema and significantly decreased TBARS, H_2_O_2_, TNF-α, and IL-6 levels in rat kidneys. α-LA also resulted in increase −SH group, total protein, and SOD levels and ameliorated the GSH redox status when compared to the LPS group. The results suggest that α-LA plays an important role against LPS-induced oxidative stress in kidney tissue as well as downregulating the expression of pro-inflammatory cytokines.

**Graphical Abstract:**

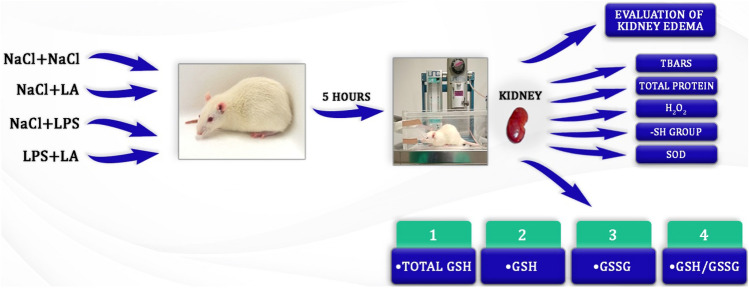

## Introduction

Oxidative stress destabilizes the physiological processes of homeostasis, thus damaging various biomolecules, such as lipids, proteins and DNA and the consequent disruption of their function Oxidative stress is caused by an imbalance between the quenching and production of oxygen free radicals. It is responsible for damage of mitochondria, causing mitochondrial malfunctions and slowing tissue regeneration (Hussain et al. 2022).

Oxidative stress can also lead to chronic inflammation. It was reported to elevate the level of pro-inflammatory factors such as tumour necrosis factor (TNF)-α, interleukin (IL)-6, and IL-1 by affecting monocytes and macrophages. The overexpressed pro-inflammatory cytokines may induce a pathological response and leads to organ dysfunction (Tejchman et al. 2021). Such stress can lead to multiorgan failure and promote the formation of many clinical disorders, such as kidney and cardiovascular diseases, atherosclerosis, heart attacks, strokes, neoplastic diseases, neurodegenerative disease, diabetes, degenerative diseases of bones and joints, infertility, asthma and aging (Alahmar 2019; Ansari et al. 2020; Hayes et al. 2020; Kattoor et al. 2017; Liu et al. 2017; Luo et al. 2020; Mishra et al. 2018; Senoner and Dichtl 2019; Shaafi et al. 2021; Zhang et al. 2020). Kidneys are especially vulnerable to oxidative stress and damage. Oxidative stress plays the key role in the pathophysiology Acute Kidney Injury (AKI), which is characterized by an abrupt loss of kidney function (Suh et al. 2015).

Lipopolysaccharide (LPS) is a component of the cell wall of Gram-negative bacteria and is often used to induce oxidative stress in animal models (Badshah et al. 2019; Proniewski et al. 2019; Xu et al. 2022). LPS is also involved in the pathogenesis of intensified oxidative stress and can contribute to a decline in renal function (Yoo et al. 2020). Oxidative stress causes glomerular injury, microvascular dysfunction and tubular injury, and consequently, leads to AKI (Alobaidi et al. 2015; Gómez and Kellum 2016). Studies have shown oxidative stress to stimulate apoptosis in renal epithelial cells and leads to renal failure (Ow et al. 2021; Peerapornratana et al. 2019). Therefore, the development of new therapeutic agents for inhibiting the development of oxidative stress is of great clinical importance.

Multiple antioxidant defence systems exist to protect against reactive oxygen species (ROS) accumulation and thus against oxidative stress. These include non-enzymatic antioxidants, such as vitamins E and C, beta-carotene, ubiquinone, urate, reduced glutathione (GSH) and α-lipoic acid (α-LA) (Herb et al. 2021).α-LA exists in oxidized and reduced forms; the latter has a strong antioxidant effect and plays an important function in metabolism. α-LA can be synthesized through enzymatic reactions in plants and animals’ mitochondria from octanoic acid and cysteine (as a sulphur donor). Mammalian cells can synthesize α-LA through the action of mitochondria lipoic acid synthase which can be downregulated in different clinical conditions. α-LA is found in meats and vegetables such as spinach, broccoli, tomato. It is also available as a dietary supplements. After orally taken, α-LA is absorbed by intestine and is transported to various organs, including the brain (it has the potential to cross freely the blood–brain barrier) (Shay et al. 2009). Independently from sources (diet or nutritional supplements), α-LA, it is transformed to DHLA (reduced form, hydrolipidic acid) and metabolized in the liver to various metabolites, such as bisnorliponate and tetranorliponate, and has the kidneys excretion. There are some systems have been related with the cellular transport of α-LA, such as sodium-dependent transport, a transmembrane protein produced by the SLC5A6 gene, which also translocated other vitamins and cofactors. Both transporters are also responsible for α-LA intestinal uptake (Vadlapudi et al. 2012).

Chemically, α-LA (1,2 dithiolane-3-pentanoic acid) is a disulphide derivative of octanoic acid that forms an intramolecular disulphide bond in its oxidized form. The two sulphur atoms in the 1,2-dithiolane ring confers upon α-LA a high tendency for reduction of other redox-sensitive molecules (Zhang et al. 2016). α-LA is used as a co-factor of the mitochondrial respiratory enzymes pyruvate dehydrogenase and α-ketoglutarate dehydrogenase (Cronan 2020).

In addition, a large body of literature data indicates the effectiveness of α-LA in the prevention of oxidative stress-mediated pathologic conditions, such as brain disease and cognitive dysfunction, obesity, cardiovascular disease, hypertension, certain types of cancer, glaucoma, osteoporosis, diabetes and related complications, such as retinopathy, nephropathy, neuropathy, wound healing and cardiovascular diabetic autonomic neuropathy (Seifar et al. 2019; Theodosis-Nobelos et al. 2021). The antioxidant effects of α-LA were found in rats subjected to sepsis in which α-LA was able to decrease oxidative stress in the kidney and other organs, including the liver and heart (Petronilho et al. 2016). Additionally, the studies demonstrated that a naturally occurring cellular antioxidant α-LA acts as a free radical scavenger of ROS and reactive nitrogen species for renoprotection. The mechanisms whereby α-LA exerts its protective effects are not clear but are probably related to the phosphatidylinositol 3-kinase/Akt/Nrf2 pathway and the PI3-kinase/Akt pathways (Zhang et al. 2016). In addition, α-LA demonstrates anti-inflammatory properties by downregulating the expression of redox-sensitive pro-inflammatory proteins, including TNF-α, IL-6, and inducible nitric oxide synthase (Moura et al. 2015). α-LA may also take part in the suppressing of overproduction of endothelin 1, the peptide that contributes to the development of inflammatory processes in the vascular wall of the renal blood vessels.

Because of its anti-inflammatory and antioxidant abilities, α-LA could be used in the therapy of oxidative stress induced kidney diseases, and may be a promising strategy for preventing the progression of kidney damage.

Based on the suppression of oxidative stress and improvement of inflammation parameters, this study provides a new evidence that α-LA protects kidneys from oxidative stress and inflammation.

## Materials and Methods

### Animals and Treatments

Male Wistar rats aged 2–3 months, weighing 260–280 g were used for the experiment. The animals were housed 3–4 per cage in standard laboratory conditions: 12 h light/dark cycles at a temperature of 22 ± 2 °C and 55 ± 5% air humidity. Rats were allowed free access to water ad libitum and a standard laboratory diet (LSM, supplied by the Agropol, Poland). After 1 week of acclimatization, the rats were randomly assigned to four groups (*n* = 8), as follows:

Group I—NaCl (control); received two doses of 0.2 mL 0.9% NaCl, 0.5 h apart;

Group II—LA; received 0.2 mL 0.9% NaCl and 0.5 h later received α-LA (60 mg/kg b.w.);

Group III—LPS; received 0.2 mL 0.9% NaCl and 0.5 h later received LPS (30 mg/kg b.w.);

Group IV—LPS + LA; received LPS (30 mg/kg) and 0.5 h later received α-LA (60 mg/kg b.w.).

The rats were weighed before and after the experiment. After the administration of α-LA or LPS, each group of animals was observed for a period of 5 h.

All compounds were injected into the tail vein and after 5 h, the animals were anaesthetized with a mixture of ketamine (87 mg/kg) and xylazine (13 mg/kg) injected intramuscularly. Then, the animals were euthanized by decapitation.

The reagents were provided by Sigma Aldrich Chemical Co. (St. Louis Mo, USA) or POCH (Gliwice, Poland). α-Lipoic acid and lipopolysaccharide from *Escherichia coli* (LPS 026:B6), lyophilized powder chromatographically purified by gel filtration (protein content < 1%) were purchased from Sigma Aldrich Chemical Co. (St. Louis Mo, USA). The commercial rat kits were used to evaluate the thiobarbituric acid reactive substances (TBARS), superoxide dismutase (SOD), total, reduced, and oxidized glutathione and GSH/GSSG ratio assays.

The kidneys were removed, rinsed with cold isotonic saline and stored at − 80 °C for preparation homogenates for biochemical assessment oxidative stress markers.

### Determination of Oxidative Stress Biomarkers

#### Determination of TBARS Concentration

TBARS were measured in kidney tissue using test Cayman Chemical TBARS Assay Kit (Item No. 10009055-96, USA), following the manufacturer’s instructions.

Twenty five mg portions of kidney tissue were cut into small pieces and homogenized in 250 μL of ice-cold RIPA Buffer with protease inhibitors in homogenizer. The homogenates were centrifuged for 10 min at 1600 ×*g* in 4 °C. After centrifuging, the obtained supernatants were analysed.

The optical density was measured in a spectrophotometer at 532 nm. Data were expressed as μM.

#### Determination of Tissue H_2_O_2_ Content

Hydrogen peroxide (H_2_O_2_) generation in homogenates was measured using horseradish peroxidise/homovanillic acid (HRP/HVA) systems according to Ruch et al. (1983). In brief, 50 mg of tissue were homogenized with 2 mL of 1.15% KCl. Then, 10 μL homogenate was divided between two Eppendorf tubes. One tube received a reaction mixture for the calibration curve, which contained phosphate-buffered saline (PBS; pH 7.0) and HRP (1 U/mL) containing 400 μmol HVA. The second tube received PBS and 1 U/mL HRP.

Both tubes were incubated for 60 min at 37 °C. Then, PBS and 0.1 M glycine–NaOH buffer (pH 12.0) with 25 mM EDTA were added to each tube to stop the enzymatic reaction. Excitation was set at 312 nm and emission was measured at 420 nm (Perkin Elmer Luminescence Spectrometer, LS-50, Norwalk, CT, USA). Readings were converted into H_2_O_2_ concentration using the regression equation prepared from three series of calibration experiments with ten increasing H_2_O_2_ concentrations (range 10–1000 μM).

#### Determination of Sulfhydryl Groups (−SH) Levels

Free sulfhydryl groups were determined using Ellman’s method (Ellman 1970). Ellman’s reagent (DTNB; 5,5′-dithiobis-(2-nitrobenzoic) acid) served as a chromogen to measure the thiol levels.

Fifty mg portions of kidney tissue were cut into small pieces and homogenized in 2 mL of ice-cold 0.1 M PBS. The homogenates were centrifuged for 10 min at 5000 ×*g* at 4 °C. Then, the Ellman’s reagent was added to the above supernatant.

The absorbance of the obtained solution was measured at 412 nm using a LS-50 Luminescence Spectrometer (Perkin Elmer, Norwalk, USA). Concentrations of −SH groups were expressed as μM.

#### Determination of Total Protein

The determination of total protein was assessed according to Lowry et al. (1951) using bovine serum albumin (BSA) as standard.

Fifty mg portions of kidney tissue were cut into small pieces and homogenized in 2 mL of ice-cold 0.1 M PBS. The homogenates were centrifuged for 10 min at 5000 ×*g* at 4 °C with protease inhibitors.

To 0.1 mL of the sample or standard (10, 20, 40, 80, and 100 μg/mL), was added 0.1 mL of 2 N NaOH and hydrolyzed at 100 °C for 10 min in a boiling water bath. Hydrolysate was allowed to cool down, and 1 mL of freshly mixed complex-forming reagent (2% (*w*/*v*) Na_2_CO_3_ in distilled water + 1% (*w*/*v*) CuSO_4_·5H_2_O in distilled water + 2% (*w*/*v*) sodium potassium tartrate in distilled water) was added to it. The solution was maintained at room temperature for 10 min. Then, 0.1 mL of Folin reagent was added to the above solution, and the mixture was maintained at room temperature for 50 min.

The values for absorbance were read at 700 nm (Pharmacia LKB Ultraspec III UV/VISIBLE spectrophotometer). The values for absorbance were converted into protein concentration using a standard curve for ten increasing BSA concentrations (5–250 μg/mL).

#### Determination of SOD Activity

Kidney tissue SOD activity was measured using a commercially available ELISA test kit containing a monoclonal antibody specific for rat SOD (Cloud-Clone Corp., USA). The procedure was performed according to the manufacturer’s instructions.

Fifty mg portions of kidney tissue were cut into small pieces and homogenized in 1 mL of fresh lysis buffer. The resulting suspension was sonicated with an ultrasonic cell disrupter till the solution is clarified. Then, the homogenates were centrifugated for 5 min at 10,000 ×*g*.

The values for absorbance were read at 450 nm (Pharmacia LKB Ultraspec III UV/VISIBLE spectrophotometer). SOD activity was expressed as ng/mg protein.

#### Determination of Total Glutathione (tGSH), Reduced Glutathione (GSH), Oxidized Glutathione (GSSG), and GSH/GSSG Levels

Total glutathione was measured using the Cayman Chemical Glutathione Assay kit (Item No.703002-96, Ann Arbor, USA). The GSH, GSSG, and GSH/GSSG ratio were analysed using the Abnova GSH/GSSG Assay kit (Item No. KA3779, USA), following to the manufacturer’s instructions.

To measure total (GSH total) and oxidized state (GSSG) of glutathione, tissue sample was divided into two parts. Both parts were homogenized in the same way (in buffer containing 50 mM phosphate and 1 mM EDTA).The only difference was the addition of a Scavenger (2-vinylpyridinium) to the GSSG sample. Scavenger complexes with GSH and prevents it from reacting with the dye and interfering with the measurement of GSSG.

The calculation of total, oxidized and reduced states of glutathione were determined according to the manufacturer’s instructions.

#### Determination of TNF-α and IL-6

The commercial rat ELISA kit (R&D Systems, USA) was used to evaluate TNF-α, and for IL-6 the Cloud-Clone Corp., High Sensitive Enzyme-linked Immunosorbent Assay Kit (HEA079Ra, USA) was used.

The procedures were performed according to the manufacturer’s instructions.

Fifty mg portions of kidney tissue were cut into small pieces and homogenized in 2 mL of ice-cold PBS with protease inhibitors. The resulting suspension was subjected to two freeze–thaw cycles to further break cell membranes. The homogenates were centrifuged for 5 min at 5000 ×*g* in 4 °C. After centrifuging, the supernatant was separated and applied to determine TNF-α and IL-6.

#### Determination of Kidney Oedema

The kidneys were rinsed with cold 0.9% NaCl, dried, and weighed to estimate kidney oedema. All the rats weighed during the experiment. The ratios of kidney weight to body weight (KW/BW) were measured, and used as an index of kidney oedema.

### Ethics

The study complied with the Guide for the Care and Use of Laboratory Animals published by the U.S. National Institutes of Health (8th Ed., 2011; Bethesda, MD, USA) and met the approval of the Ethics Committee of the Medical University of Lodz, Poland permission number 7/LB699/2014 and was conducted according to guidelines of the Declaration of Helsinki.

### Statistical Analysis

Statistical analyses were conducted with the use of StatSoft software (version StatSoft version 1.0.67. www.statsoft.pl). The normal distribution of data was checked by using the Shapiro–Wilk test. The Leven test was used to check the homogeneity of variance. One-way ANOVA with the post hoc Duncan’s multiple range test was used to compare the means of multiple groups, because the data met the normal distribution with homogeneity of variance. The level of statistical significance was defined as *P* < 0.05. The data are presented as mean ± SD from eight animals in each group.

## Results

### Observed Changes in Behaviour After LPS/LA Administration

LPS caused behavioural alterations in rats such as appetite alterations, dysregulation of the motion system, and anxiety symptoms, among others. Moreover, the rats were a number of unpleasant symptoms such as weakness, reduced mobility and sore all body. They presented lower ambulation when placed in cage. Neurophysiological symptoms also included irritable moods and anxiety. LPS acts as a stressor causing the production of excess free radicals. High free radicals levels may increase the brains vulnerability to oxidative stress which may have adverse effects on neurobehavioral health.

In contrast, the administration of LA after LPS caused decreased neurophysiological symptoms. The rats became less irritable and more active.

The body weight of the rats during the study did not change significantly in experimental groups. There was only a slight decrease in body weight in the LPS group compared to control.

### Effects of Oxidative Stress Biomarkers

#### TBARS Evaluation

**Fig. 1:**
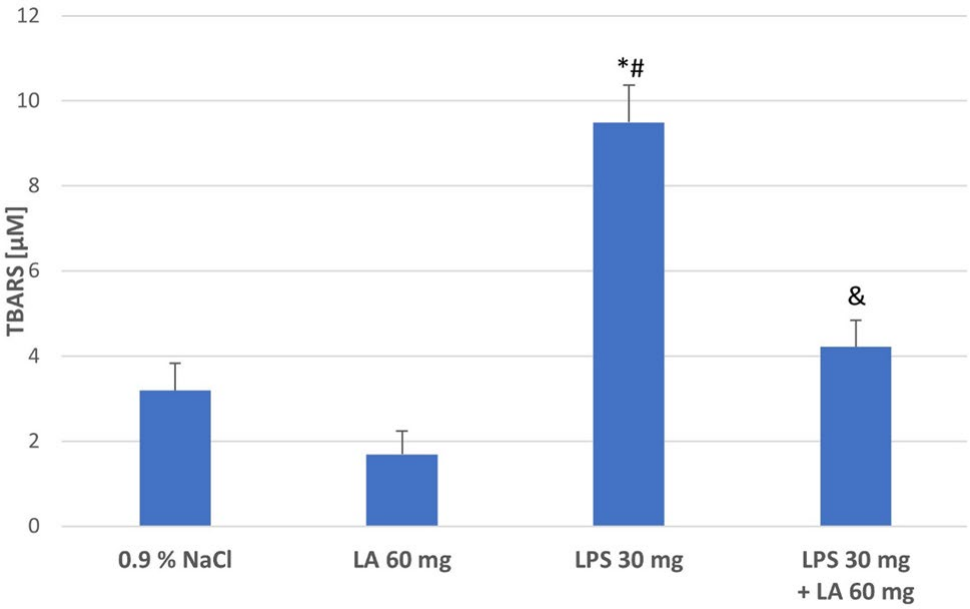
The effect of LA, LPS, and LPS + LA on TBARS levels in kidney homogenates. Results are shown as means ± SD, *n* = 8. ^*^P < 0.05 vs. NaCl; ^#^*P* < 0.01 vs. LA; ^&^*P* < 0.05 vs. LPS. For normal distribution: Shapiro–Wilk test; One-way ANOVA showed significant differences in TBARS concentrations between different treatment groups (*F* = 33578.14; *df* = 3; *P* < 0.001).

The amount of lipid peroxidation of the kidney homogenates was estimated by measuring TBARS. The TBARS levels were raised dramatically following LPS administration compared to the Controls (*P* < 0.05) The TBARS level in the LPS + LA group was statistically significantly lower than in the LPS group (*P* < 0.05). Treatment with α-LA normalized kidney TBARS levels to Control levels (see Fig. 1). These data suggest that α-LA lessens LPS-induced oxidative stress in rats’ renal tissues.

#### Effect of LA on LPS-Induced H_2_O_2_ Level

**Fig. 2:**
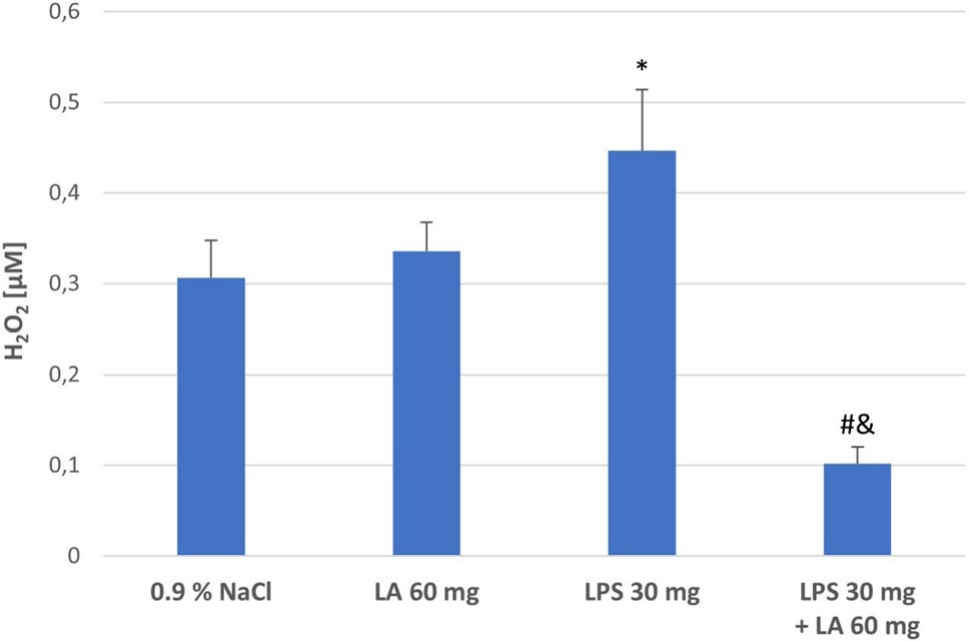
The effect of LA, LPS, and LPS + LA on H_2_O_2_ generation in kidney homogenates. Results are shown as means ± SD, *n* = 8. *P < 0.01 vs. NaCl, LA; ^#^*P* < 0.05 vs. LPS; ^&^*P* < 0.05 vs. NaCl, LA. For normal distribution: Shapiro–Wilk test; One-way ANOVA showed significant differences in H_2_O_2_) concentrations between different treatment groups (*F* = 4516.13; *df* = 3; *P* < 0.001)

To investigate the endogenous generation of ROS, H_2_O_2_ was measured in rat kidney homogenates. The results showed that the H_2_O_2_ concentrations in the kidney homogenates were increased in the LPS group compared with the Controls (*P* < 0.01). The treatment of α-LA significantly decreased H_2_O_2_ concentrations (*P* < 0.05; see Fig. 2).

#### Assessment of Sulfhydryl Groups (−SH)

**Fig. 3:**
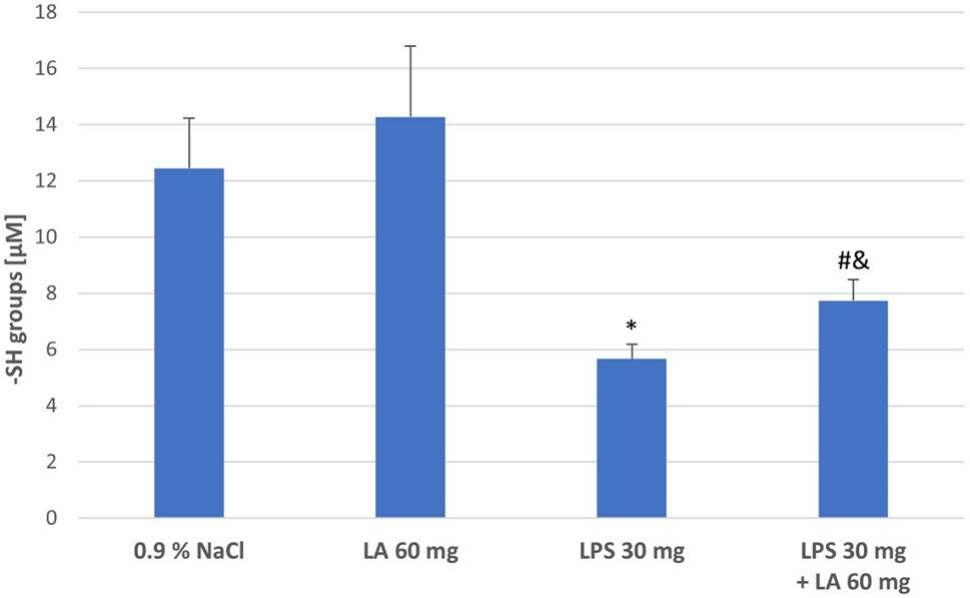
The effect of LA, LPS, and LPS + LA on sulfhydryl groups (−SH) content in kidney homogenates. Results are shown as means ± SD, *n* = 8. ^*^P < 0.05 vs. NaCl, LA; ^#^*P* < 0.05 vs. LPS; ^&^*P* < 0.01 vs. NaCl, LA. For normal distribution: Shapiro–Wilk test; One-way ANOVA showed significant differences in −SH concentrations between different treatment groups (*F* = 10394.31; *df* = 3; *P* < 0.001)

Oxidative damage −SH groups was measured to determine loss of protein biological activity. The levels of total −SH groups in kidney homogenates after administration of LPS decreased compared to the Controls and LA groups (*P* < 0.05). Administration of α-LA after LPS infusion result in a significant increase in −SH groups levels compared to the LPS group (*P* < 0.05; see Fig. 3).

#### Effect of LA on Total Protein

**Fig. 4:**
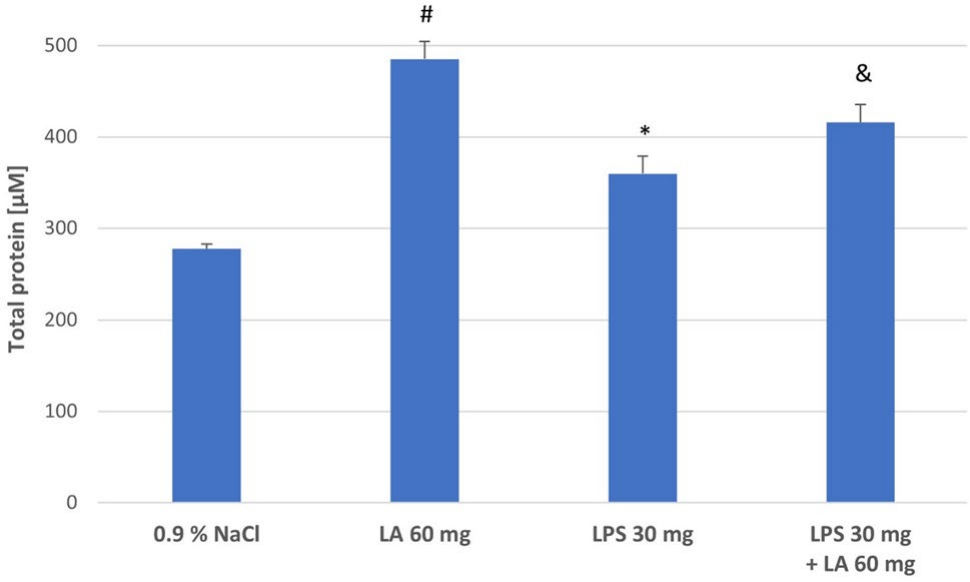
The effect of LA, LPS, and LPS + LA on total protein in kidney homogenates. Results are shown as means ± SD, *n* = 8. ^*^P < 0.05 vs. LA; ^#^*P* < 0.05 vs. NaCl; ^&^*P* < 0.05 vs. LPS; ^#^*P* < 0.05 vs. LPS. For normal distribution: Shapiro–Wilk test; One-way ANOVA showed significant differences in total protein concentrations between different treatment groups (*F* = 439.82; *df* = 3; *P* < 0.001)

To investigate the protein oxidation, total protein was measured in rat kidney homogenates. Consistent with the results reported, the kidney homogenates in LPS group exhibited lower total protein content than the kidney homogenates in LA group (*P* < 0.05). The concentration of total protein increased after administration of *α-*LA (*P* < 0.05; see Fig. 4).

#### Effect of LA on SOD Activities

**Fig. 5:**
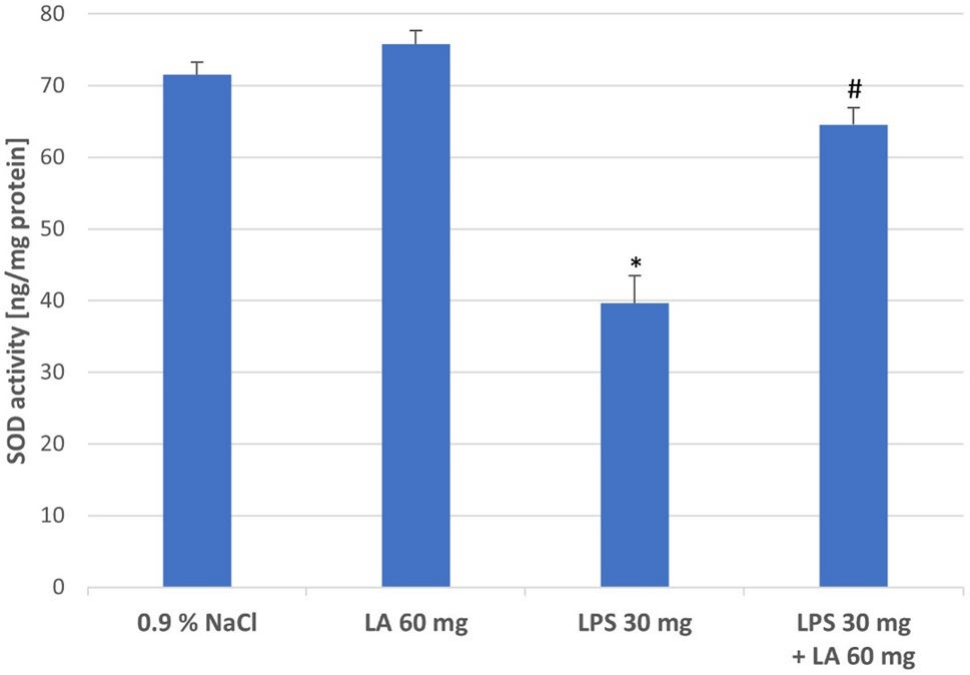
The effect of LA, LPS, and LPS + LA on SOD activities in kidney homogenates. Results are shown as means ± SD, *n* = 8. *P < 0.05 vs. NaCl, LA; ^#^*P* < 0.05 vs. LPS. For normal distribution: Shapiro–Wilk test; One-way ANOVA showed significant differences in SOD concentrations between different treatment groups (*F* = 937.11; *df* = 3; *P* < 0.0001)

As shown in Fig. 5, significant decreased activities of SOD was found in kidney homogenates after LPS administration, which is interpreted as a worsening of oxidative stress (*P* < 0.05). While α-LA was effective in increasing SOD activity in the kidney homogenates, which indicates stronger antioxidant defence (*P* < 0.05).

#### Assessment of Glutathione

Table 1 shows the levels of tGSH, GSH, GSSG, and GSH/GSSG ratio in kidney homogenates of the experimental groups of rats.

**Table 1:** Kidney status of glutathione metabolism in the experimental groups

Parameters	NaCl	LA	LPS	LPS + LA
Total GSH (µM)	82.8 ± 13.7	77.6 ± 47.9	12.5 ± 5.6**	93.8 ± 39.1*
GSH (µM)	69.5 ± 16.7	56.2 ± 13.8	4.9 ± 5.6**	89.9 ± 14.7*
GSSG (µM)	19.8 ± 8.2	5.7 ± 2.7^&^	18.9 ± 5.8^##^	19.1 ± 3.4
GSH/GSSG (redox status)	3.0 ± 2.1	5.9 ± 1.3	0.2 ± 0.3^#^^,^^**^	3.9 ± 1.2*

Values are expressed as mean ± SD, *n* = 8 in each group. One-way ANOVA showed significant differences in total GSH, GSH, GSSG and GSH/GSSG concentrations between different treatment groups (*F* = 527.54; *F* = 2009.38; *F* = 650.08; *F* = 1034.78, respectively; *df* = 3; *P* < 0.001)

*P < 0.05 vs. LPS;

**P < 0.01 vs. NaCl, LA;

^#^*P* < 0.05 vs. NaCl;

^##^*P* < 0.001 vs. LA;

^&^*P* < 0.01 vs. NaCl

In the LPS group, the GSH level decreased significantly compared to the NaCl and LA groups (*P* < 0.01) and was associated with an increase in GSSG (*P* < 0.001). The GSH/GSSG ratio, which reflects the intracellular glutathione redox balance, also decreased significantly in the LPS group compared to the NaCl and LA groups (*P* < 0.05, *P* < 0.01 appropriately) indicating a weakening of the antioxidative potential. In the LPS + LA group, both the reduced GSH level and the GSH/GSSG ratio increased compared to the LPS group (*P* < 0.05).

Treatment with LA restored the kidney antioxidant defence, indicated by the GSH/GSSG ratio.

#### Assessment of TNF-α and IL-6

As shown in Table 2, the LPS administration caused a marked increase in the levels of TNF-α and IL-6 in kidney homogenates compared to Control and LA group (*P* < 0.05). TNF-α and IL-6 levels appeared to be significantly reversed after *α-*LA treatment (*P* < 0.01).

**Table 2:** Effect of α-LA, LPS and their combination on TNF-α and IL-6 levels in kidney homogenates

Parameters	NaCl	LA	LPS	LPS + LA
TNF-α (pg/mL)	34.7 ± 21.2	42.2 ± 28.1	188.3 ± 47.3*,#	96.4 ± 23.2**,&
IL-6 (pg/mL)	27.7 ± 9.1	33.5 ± 11.1	168.4 ± 40.8*,#	83.2 ± 20.1**,&

Values are expressed as mean ± SD, *n* = 8 in each group. One-way ANOVA showed significant differences in TNF-α and IL-6 concentrations between different treatment groups (*F* = 1437.14 and *F* = 1684.47, respectively; *df* = 3;* P* < 0.001)

*P < 0.001 vs. Control;

**P < 0.05 vs. LPS;

^#^*P* < 0.01 vs. LA

#### Changes in the KW/BW Ratio in the NaCl, LA, LPS, and LPS + LA Groups

The KW/BW ratio was significantly higher in the LPS group compared to the Controls *and LA groups (P* < 0.05). Administration of *α-*LA reduced the increases in KW/BW ratio, which suggest that *α-*LA attenuated kidney oedema (*P* < 0.01; see Table 3).

**Table 3:** Changes in the KW/BW ratio in the experimental groups

Group	KW/BW (mg/g)
NaCl	2.7 ± 0.6
LA	2.9 ± 0.5
LPS	4.4 ± 1.1*
LPS + LA	3.5 ± 0.9^#^

One-way ANOVA showed significant differences in KW/BW concentrations between different treatment groups (*F* = 127.21; *df* = 3; *P* < 0.001)

*P < 0.05 vs. NaCl, LA; *P* < 0.01 vs. LPS. Values are expressed as mean ± SD, *n* = 8 in each group

## Discussion

Kidney diseases are a serious health problem. Very often, they are caused by oxidative stress, which damages kidney tissue and promotes inflammation leading to further tissue damage with the accumulation of impaired biomacromolecules. Many investigations, carried on animal models, have utilized systemic administration of α-lipoic acid for estimation of the protective effects of α-LA against kidney injury. Despite the great interest in this issue, there is still a little research demonstrating the effects of α-lipoic acid after inducing AKI. Thus, the main objective was to investigate the protective effect of α-LA on LPS-induced oxidative kidney damage and inflammation and to elucidate the molecular mechanisms involved in this protective effect.

Oxidative stress exists in many diseases of the renal apparatus such as glomerulonephritis and tubulointerstitial nephritis, renal failure, uraemia and proteinuria, and is a common cause of damage to the interstitial tissue of the kidneys and kidney cells (Pizzino et al. 2017). The redox imbalance results from the overproduction of ROS, which is formed in the kidney mainly in the mitochondrial respiratory chain and as a result of NADPH oxidase activity. Moreover, mitochondrial dysfunction and ROS generation caused by oxidative stress may lead to loss of mitochondrial membrane potential and activation of the mitochondrial apoptotic pathway, which is particularly important in the pathogenesis of chronic kidney disease (Locatelli et al. 2003). Due to the presence of a large number of mitochondria in the kidney, this organ is particularly vulnerable to be damaged by oxidative stress (Sureshbabu et al. 2015).

Many of the protective mechanisms against excessive free radical production and oxidative stress employ antioxidants with are one of the most effective α-LA (Sharifi-Rad et al. 2020). The multidirectional biological effect of α-LA contributes to the inhibition of oxidative stress and thus to the reduction of the amount of ROS. Therefore, α-LA is effective in the treatment of many free radical diseases, as was mentioned previously. Some studies also indicate that α-LA can protect against oxidative stress in kidney tissue e.g. after folic acid or gold nanoparticles administration (Abdelhalim et al. 2020; Li et al. 2021).

Our previous data indicated that α-LA can protect against LPS-induced oxidative stress and inflammation in rat organs, such as the heart, spleen, liver or skeletal muscles (Gorąca et al. 2013, 2015; Kowalczyk et al. 2016; Skibska et al. 2022). The present investigation analyzed oxidative stress, induced by LPS in rat kidneys. LPS injection is the most reproducible and simple model as it allows exploring of basic processes such as inflammation and oxidative damage by bypassing bacteria-related factors, such as toxins production. Moreover, the LPS model is more relevant for sepsis-like conditions. Also, bacterial toxins administration could dramatically affect the flow and outcome of the pathology. Since the aim of our work was to address oxidative damage and antioxidant capacities in kidney homogates, we studied LPS-mediated AKI and revealed that oxidative damage can be reduced after α-lipoic acid administration. The administration of α-LA alleviated LPS-induced oxidative stress via reduction of thiobarbituric acid reactive substances (TBARS) and hydrogen peroxide (H_2_O_2_) levels. We also noted increase of total sulfhydryl groups ( −SH), total protein content, tGSH, reduced glutathione (GSH), and the activity of antioxidant enzymes such as SOD. Kidney oedema was also assessed. Kidney oedema is an indicator of inflammatory and toxicity, leading to kidney dysfunctions. Remarkable reductions in kidney oedema were noted in the α-LA-treated rats after earlier LPS administration, whereas in the LPS group, kidney oedema was aggravated relative to the controls.

Most known free radical process is the peroxidation of lipids, in which the oxidation of unsaturated acids of fatty acids proceeds with the formation of these compounds. Lipid peroxidation is pro- non-enzymatic process. Multi-saturated fatty acids due to their structure react easily with ROS. In the present study, an increase in TBARS concentration was noted after LPS administration, whereas α-LA caused a decrease in TBARS concentration compared to the LPS group. This is indicated that LPS elevates oxidative stress in the kidney, which may be due to the formation of ROS leading to lipid peroxidation (El-Desoky et. al. 2020). Ilçe et al. (2019) reported similar mechanism reaction on LPS treatment of kidney tissues, observing enhanced lipid peroxidation by increased of malondialdehyde (MDA) level.

Moreover, LPS may enhance peroxidation, interacting directly with the cellular plasma membrane and membrane polyunsaturated fatty acids (PUFAs). In the effect, by oxidizing membrane PUFAs and intracellular macromolecules, ROS cause cellular dysfunction and tissue damage (Su et al. 2019).

However, α-LA demonstrated a protective effect on renal tissue and the possibility of reversing the negative effect of ROS. Previous studies have also reported similar effects by α-LA in relation to lipid peroxidation (Abdelhalim et al. 2020; Hussein et al. 2012).

Similarly, higher concentrations of H_2_O_2_ were noticed in LPS-treated rats compared to controls; these may be due to the formation of ROS including hydrogen peroxide, superoxide anion, and hydroxyl radicals. The results in this study demonstrated that treatment of α-LA significantly decreased H_2_O_2_ concentrations in kidney tissues. The reports of other authors have suggested that H_2_O_2_ causes lipid peroxidation, markedly increasing cell permeability, impairs the cellular antioxidant defence and induces cell apoptosis. In addition H_2_O_2_ activates NADPH oxidase pathway, NADPH oxidase can transfer NADPH electrons to molecular oxygen to produce superoxide anions, indicating that ROS production is on excessive, the balance of the oxidative stress system is disturbed and eventually damages cells or tissues (Hao et al. 2016). It was also found that α-LA protects against oxidative damage induced by H_2_O_2_ and may potentially limit excessive ROS production and protect cells from peroxide toxicity (Wang et al. 2020).

Another examined factor, free −SH group content, decreased after LPS administration and increased in the renal homogenates after α-LA treatment. The lower level of free −SH groups in LPS-treated rats may be the result of elevated ROS levels in the kidney homogenates and indicated on toxic effect LPS administration. In contrast to this observation, α-LA administration entailed higher amounts of free −SH groups. Such antioxidant properties of α-LA have also been noted by other authors (Martinelli et al. 2021; Rochette et al. 2013). Additionally in this study, the nephroprotective effect of α-LA was confirmed by the analysis of changes in total protein concentration in kidney homogenate after administration of LPS. Our study is consistent with previous investigations. It has been shown that administration of α-LA in obstructive nephropathy slows down the process of kidney fibrosis and allows to maintain its partial functionality. In this case, the nephroprotective effect of α-LA was attributed to its inhibition of transforming growth factor (TGF)-β1-induced fibrogenesis. TGF-β1 is a potent profibrotic factor that stimulates the production of ROS. The strong antioxidant properties of α-LA made it possible to limit the damage to the cellular structures of the kidneys caused by excess ROS (Wongmekiat et al. 2013).

ROS have been found to oxidize thiol groups, which in turn can influence the structure and function of numerous proteins (Baba and Bhatnagar 2018). The main intracellular thiol antioxidant is the tripeptide glutathione (GSH). Decreased GSH levels result in increased ROS (Ghezzi 2011), which was also noticed in this study. α-LA increases the concentration of the oxidized form of GSH in cells, which prevents the formation of oxidative stress. Both these processes, viz. a decrease in GSH level by LPS-induced oxidative stress and the increase of the oxidized form of GSH after α-LA administration, were also noticed in this study.α-LA increases the level of intracellular glutathione in various types of cells and tissues. It increases the amount of glutathione in the cell, acting as a transcription factor inducing genes responsible for the synthesis of glutathione. α-LA increases the activity of glutamate-cysteine ligase, the enzyme catalysing the first of the two stages of GSH synthesis, resulting in its intensification. During the reduction of α-LA to DHLA, cystine is reduced to cysteine. Cysteine is a substrate which availability depends on the GSH biosynthesis process, and its greater availability enables the synthesis of larger amounts of GSH (Gomes and Negrato 2014; Rochette et al. 2015).

Furthermore, many studies indicate a role for GSH, as well as its oxidized form (GSSG), in the regulation of cellular function and gene expression, activities that go well beyond the free radical scavenger action commonly associated with GSH. Importantly, GSH participates in the redox regulation via the formation of mixed disulphides between protein cysteines and glutathione. This process, commonly referred to as glutathionylation, is known to regulate signaling proteins and transcription factors (Ghezzi 2013).

Observed effect of the increases of glutathione amount in rat cells is probably related to capacity of α-LA to regulate GSH biosynthesis via activation of transcription factor inducing genes responsible for the increase of glutathione synthesis. It was evidenced that α-LA can raise the level of key transcription factor—nuclear Nrf2 (nuclearerythroid2-related factor), and allows its release and transport to the nucleus of the cell. Nrf2 factor mediates the expression of antioxidant and detoxification genes regulated by the antioxidant response element (ARE). Nrf2 factor, in association with small Maf and Jun protein family, forms an upstream transcriptional complex, that binds to the ARE sequence of DNA and regulates ARE-driven genes that encode for detoxification enzymes and antioxidant proteins to strengthens the cell’s defence system against oxidative stress (Rochette et al. 2015).

Oxidants are also removed by the enzymatic defence system. Several enzymes inhibit free radical formation, some of which act directly by scavenging ROS, or indirectly by supporting other endogenous antioxidants (Sharifi-Rad et al. 2020). In this study, a significant decrease in SOD concentration after LPS administration was observed, indicating an increase in oxidative processes in kidney cells. Kitada et al. (2020) report lower Mn-SOD activity during oxidative stress resulting in diminished mitochondrial antioxidant capacity; this can impair the mitochondrial function and may lead to the development of kidney disease. Additionally, SOD has been found to be downregulated in chronic kidney disease, implying that an increase in superoxide is associated with oxidative stress in renal insufficiency (Ratliff et al. 2016).

Our results also demonstrated that α-LA treatment reduced LPS-stimulated release of inflammatory cytokines, such as TNF-α, IL-6. The favorable effect of α-LA in relation to suppress inflammation noticed other authors. Ying et al. (2011) stated that α-LA inhibits NF-κB pathway by activating the PI3-kinase/Akt pathway which result is in the suppression on NF-κB and in final effect, counteracting the pro-inflammatory process.

The mechanism underlying the therapeutic efficacy of α-LA as an antioxidant in the initiation and progression of various inflammatory diseases may be related to the reducing oxidative stress and then reducing pro-inflammatory chemical mediators (Li et al. 2015).

It is known that transcription factor NF-κB is a main regulator of the inflammatory response. NF-κB receives input from a variety of receptors and inflammatory inducers, including LPS to promote cellular responses that are proper to the stimulating inducer. NF-κB regulates the transcription of different genes that are important to the inflammatory response including chemokines, cytokines and adhesion molecules; it also regulates genes that negatively regulate its own activity. Hobbs et al. (2018) stated that the LPS-NF-κB signalling axis was noticeably active within 45 min of LPS treatment. In this time, cascade reactions including degradation of IκBα, phosphorylation of NF-κB and binding of NF-κB to consensus regulatory DNA sequences was noticed (Hobbs et al. 2018).

In the current study, the increase in TNF-α after LPS administration was most likely based on the mechanism described above.

Our results suggest that α-LA repress the LPS-induced expression of inflammatory mediators, such as TNF-α, IL-6 by inhibition nuclear translocation of NF-κB in renal tubular epithelial cells and the inhibition is independent of its antioxidant function. Both increased phosphorylation and nuclear translocation of NF-κB after LPS administration can be reduced by α-LA treatment. Suppressed NF-κB activation by α-LA can improve damaged kidney function.

The positive clinical effects of LA also can be as a result of the changes in the thiol redox status of signalling molecules.

In our experimental model, the factor causing oxidative stress was LPS, under the influence of which we noted an increase in the level of biomarkers associated with oxidative stress and inflammation. LPS induced higher expression of the pro-inflammatory cytokines be stimulated by NF-κB phosphorylation and it may be the main cause of oedema in animal models.

In addition, we found that renal oedema increased in the LPS group compared to the control group, while a significant reduction in renal oedema was observed in rats treated with α-LA after pre-administration of LPS. It showed relationship between the degree of inflammation and oedema. The LPS administration caused a marked increase in the levels of TNF-α and IL-6 in kidney homogenates compared to Control and LA group. However, TNF-α and IL-6 levels appeared to be significantly reversed after α-LA treatment. An increase in LPS-induced inflammatory factors, as a result of oxidative stress, was associated with the increase in the kidney KW/BW ratio.

Thus, the correlation between oedema and oxidative stress or inflammation during exposure to LPS, may be due to shared mechanisms of pro-inflammatory cytokines actions, and activation of signalling pathways, such as NF-κB.

In conditions of severe oxidative stress, the increased generation of ROS that interact with many vital components of cells, including lipids, nucleic acids, and proteins, causing damage to cells and leading to development of inflammatory process. Administration of α-LA significantly reduced the level of lipid and protein peroxidation, and also effectively increased the enzymatic defence system against the formation of free radicals, which proves that its antioxidant effect is based on the reduction of oxidative stress. Additionally, the reduction of oxidative stress resulted decrease of the level of pro-inflammatory factors. Thus, obtained findings indicated that reduced levels of oxidative stress markers correlate with a decrease in the release of pro-inflammatory factors.

Ziegler et al. (2022) stated that α-LA exerts its effects both in the plasma membrane and cytosol, in contrast to other antioxidants, which are either water or lipid-soluble agents. Additionally, experimental studies have proved that α-LA, besides inhibition of the generation of ROS, blocks caspase-3 invigoration, nuclear DNA degeneration, and stimulation of the receptor for advanced glycation end products (Ziegler et al. 2022). Literature data and our results indicated that α-LA may be found as potential therapeutic agent in the future especially against oxidative stress and inflammation which lead to microvascular damage and, as result, to renal and other diseases. What is more, obtained findings demonstrated that LPS administration generated oxidative stress that is the key element to induce AKI. We observed change of level of studied parameters after short time (5 h). It should be noted that the nephroprotective role of α-LA has not been completely estimated in this study, which ought to be explored in the future. For comparison, the protective effects of α-LA on chronic kidney disease should also be tested in another animal model. Moreover, comprehensive preclinical and human studies are required to evaluate the efficacy of α-LA regardless of the kidney injury models and the protective mechanisms.

### Limitation Section

It would be recommended if future research related to kidneys include the analysis, such as serum creatinine and/or urea, albuminuria and/or urinary protein, urinary kidney injury molecule-1 (KIM-1) and/or neutrophil gelatinase-associated lipocalin (NGAL) and histopathological analysis, as these are important indices for assessment of renal injury.

### Conclusions

In the present study, we concluded that LPS administration stimulated oxidative stress and inflammation in kidney tissue. Moreover, we stated that α-LA could protect the kidney tissue by downregulating the production of ROS and thus reducing oxidative stress, and decrease of inflammatory cytokines synthesis the LPS-induced. Thus, the antioxidative effect of α-LA may contribute to its therapeutic action on LPS-induced kidney damage and may be a beneficial treatment strategy for kidney dysfunction caused by oxidative stress in future. In addition, the obtained results encourage further in-depth research on the molecular mechanisms fully explaining the described effects especially since research into the use of chemical components with antioxidant properties as future prophylactic and therapeutic agents is of particular interest, as they can be more effective and safer than currently available treatments.

## Data Availability

The data that support the findings of this study are available from the corresponding author upon reasonable request.

## References

[CR1] Abdelhalim MAK, Qaid HAY, Al-Mohy YH (2020). The protective roles of vitamin E and α-lipoic acid against nephrotoxicity, lipid peroxidation, and inflammatory damage induced by gold nanoparticles. Int J Nanomed.

[CR2] Alahmar AT (2019). Role of oxidative stress in male infertility: an updated review. J Hum Reprod Sci.

[CR3] Alobaidi R, Basu RK, Goldstein SL (2015). Sepsis-associated acute kidney injury. Semin Nephrol.

[CR4] Ansari MY, Ahmad N, Haqqi TM (2020). Oxidative stress and inflammation in osteoarthritis pathogenesis: role of polyphenols. Biomed Pharmacother.

[CR5] Baba SP, Bhatnagar A (2018). Role of thiols in oxidative stress. Curr Opin Toxicol.

[CR6] Badshah H, Ikram M, Ali W (2019). Caffeine may abrogate LPS-induced oxidative stress and neuroinflammation by regulating Nrf2/TLR4 in adult mouse brains. Biomolecules.

[CR7] Cronan JE (2020). Progress in the enzymology of the mitochondrial diseases of lipoic acid requiring enzymes. Front Genet.

[CR8] El-Desoky GE, Wabaidur SM, Al Othman ZA (2020). Regulatory role of nano-curcumin against tartrazine-induced oxidative stress, apoptosis-related genes expression and genotoxicity in rats. Molecules.

[CR9] Ellman GL (1970). SH group determination in biological fluids. Anal Biochem.

[CR10] Ghezzi P (2011). Role of glutathione in immunity and inflammation in the lung. Int J Gen Med.

[CR11] Ghezzi P (2013). Protein glutathionylation in health and disease. Biochim Biophys Acta.

[CR12] Gomes MB, Negrato CA (2014). Alpha-lipoic acid as a pleiotropic compound with potential therapeutic use in diabetes and other chronic diseases. Diabetol Metab Syndr.

[CR13] Gómez H, Kellum JA (2016). Sepsis-induced acute kidney injury. Curr Opin Crit Care.

[CR14] Gorąca A, Huk-Kolega H, Kleniewska P (2013). The effects of lipoic acid on spleen oxidative stress after LPS administration. Pharmacol Rep.

[CR15] Gorąca A, Huk-Kolega H, Kowalczyk A (2015). Anti-oxidative and anti-inflammatory effects of lipoic acid in rat liver. Postępy Hig Med Dośw.

[CR16] Hao XL, Kang Y, Li JK (2016). Protective effects of hyperoside against H_2_O_2_-induced apoptosis in human umbilical vein endothelial cells. Mol Med Rep.

[CR17] Hayes JD, Dinkova-Kostova AT, Tew KD (2020). Oxidative stress in cancer. Cancer Cell.

[CR18] Herb M, Schramm M (2021). Functions of ROS in macrophages and antimicrobial immunity. Antioxidants.

[CR19] Hobbs S, Reynoso M, Geddis AV (2018). LPS-stimulated NF-κB p65 dynamic response marks the initiation of TNF expression and transition to IL-10 expression in RAW 264.7 macrophage. Physiol Rep.

[CR20] Hussain H, Ahmad S, Wadood ASS (2022). Investigation of antistress and antidepressant activities of synthetic curcumin analogues: behavioral and biomarker approach. Biomedicines.

[CR21] Hussein SA, Hassanin MR, Barky EL, AR,  (2012). Biochemical effect of alpha-lipoic acid on lipid profiles, lipid peroxidation and status of antioxidant enzymes in streptozotocin induced diabetes in rats. Benha Vet Med J.

[CR22] Ilçe F, Gök G, Pandir D (2019). Acute effects of lipopolysaccharide (LPS) in kidney of rats and preventive role of vitamin E and sodium selenite. Hum Exp Toxicol.

[CR23] Kattoor AJ, Pothineni NVK, Palagiri D (2017). Oxidative stress in atherosclerosis. Curr Atheroscler Rep.

[CR24] Kitada M, Xu J, Ogura Y (2020). Manganese superoxide dismutase dysfunction and the pathogenesis of kidney disease. Front Physiol.

[CR25] Kowalczyk A, Jeleń A, Żebrowska M (2016). BQ123 stimulates skeletal muscle antioxidant defense via Nrf2 activation in LPS-treated rats. Oxid Med Cell Longer.

[CR26] Li G, Fu J, Zhao Y (2015). Alpha-lipoic acid exerts anti-inflammatory effects on lipopolysaccharide-stimulated rat mesangial cells via inhibition of nuclear factor kappa B (NF-κB) signaling pathway. Inflammation.

[CR27] Li X, Zou Y, Fu YY (2021). A-Lipoic acid alleviates folic acid-induced renal damage through inhibition of ferroptosis. Front Physiol.

[CR28] Liu Z, Zhou T, Ziegler AC (2017). Oxidative stress in neurodegenerative diseases: from molecular mechanisms to clinical applications. Oxid Med Cell Longev.

[CR29] Locatelli F, Canaud B, Eckardt KU (2003). Oxidative stress in end-stage renal disease: an emerging threat to patient outcome. Nephrol Dial Transplant.

[CR30] Lowry OH, Rosebrough NJ, Farr AL (1951). Protein measurement with the folin phenol reagent. J Biol Chem.

[CR31] Luo J, Mills K, Cessie S (2020). Ageing, age-related diseases and oxidative stress: what to do next?. Ageing Res Rev.

[CR32] Martinelli I, Tomassoni D, Roy P (2021). Antioxidant properties of alpha-lipoic (Thioctic) acid treatment on renal and heart parenchyma in a rat model of hypertension. Antioxidants.

[CR33] Mishra V, Banga J, Silveyracd P (2018). Oxidative stress and cellular pathways of asthma and inflammation: therapeutic strategies and pharmacological targets. Pharmacol Ther.

[CR34] Moura FA, de Andrade KQ, dos Santos JC (2015). Lipoic acid: Its antioxidant and anti-inflammatory role and clinical applications. Curr Top Med Chem.

[CR35] Ow CPC, Trask-Marino A, Betrie AH (2021). Targeting oxidative stress in septic acute kidney injury: from theory to practice. J Clin Med.

[CR36] Peerapornratana S, Manrique-Caballero CL, Gómez H (2019). Acute kidney injury from sepsis: current concepts, epidemiology, pathophysiology, prevention and treatment. Kidney Int.

[CR37] Petronilho F, Florentino D, Danielski LG (2016). Alpha-Lipoic acid attenuates oxidative damage in organs after sepsis. Inflammation.

[CR38] Pizzino G, Irrera N, Cucinotta M (2017). Oxidative stress: harms and benefits for human health. Oxid Med Cell Longev.

[CR39] Proniewski B, Kij A, Sitek B (2019). Multiorgan development of oxidative and nitrosative stress in LPS-induced endotoxemia in C57Bl/6 mice: DHE-based in vivo approach. Oxid Med Cell Longev.

[CR40] Ratliff BB, Abdulmahdi W, Pawar R (2016). Oxidant mechanisms in renal injury and disease. Antioxid Redox Signal.

[CR41] Rochette L, Ghibu S, Richard C (2013). Direct and indirect antioxidant properties of-lipoic acidand therapeutic potential. Mol Nutr Food Res.

[CR42] Rochette L, Ghibu S, Muresan A (2015). Alpha-lipoic acid: molecular mechanisms and therapeutic potential in diabetes. Can J Physiol Pharmacol.

[CR43] Ruch W, Cooper PH, Baggiolinii M (1983). Assay of H_2_O_2_ production by macrophages and neurotrophils with homovanillic acid and horse-radish peroxidase. J Immunol Methods.

[CR44] Seifar F, Khalili M, Khaledyan H (2019). α-Lipoic acid, functional fatty acid, as a novel therapeutic alternative for central nervous system diseases: a review. Nutr Neurosci.

[CR45] Senoner T, Dichtl W (2019). Oxidative stress in cardiovascular diseases: still a therapeutic target?. Nutrients.

[CR46] Shaafi S, Hadisi F, Mahmoudinezhad M (2021). The significance of the oxidative stress markers in the one-year prognosis of patients with acute ischemic stroke: a case-control study. BMC Neurol.

[CR47] Sharifi-Rad M, Kumar NVA, Zucca P (2020). Lifestyle, oxidative stress, and antioxidants: back and forth in the pathophysiology of chronic diseases. Front Physiol.

[CR48] Shay KP, Moreau RF, Smith EJ (2009). Alpha-lipoic acid as a dietary supplement: molecular mechanisms and therapeutic potential. Biochim Biophys Acta.

[CR49] Skibska B, Goraca A, Skibska A (2022). Effect of alpha-lipoic acid on rat ventricles and atria under LPS-induced oxidative stress. Antioxidants.

[CR50] Su LJ, Zhang JH, Gomez H (2019). Reactive oxygen species-induced lipid peroxidation in apoptosis, autophagy, and ferroptosis. Oxid Med Cell Longev.

[CR51] Suh SH, Lee KE, Kim IJ (2015). Alpha-lipoic acid attenuates lipopolysaccharide-induced kidney injury. Clin Exp Nephrol.

[CR52] Sureshbabu A, Ryter SW, Choi ME (2015). Oxidative stress and autophagy: crucial modulators of kidney injury. Redox Biol.

[CR53] Tejchman K, Kotfis K, Sieńko J (2021). Biomarkers and mechanisms of oxidative stress-last 20 years of research with an emphasis on kidney damage and renal transplantation. Int J Mol Sci.

[CR54] Theodosis-Nobelos P, Papagiouvannis G, Tziona P (2021). Lipoic acid: kinetics and pluripotent biological properties and derivatives. Mol Biol Rep.

[CR55] Vadlapudi AD, Vadlapatla RK, Mitra AK (2012). Sodium dependent multivitamin transporter (SMVT): a potential target for drug delivery. Curr Drug Targets.

[CR56] Wang W, An LP, Li YF (2020). Alpha-lipoic acid ameliorates H_2_O_2_-induced human vein endothelial cells injury via suppression of inflammation and oxidative stress. Biosci Biotechnol Biochem.

[CR57] Wongmekiat O, Leelarungrayub D, Thamprasert K (2013). Alpha-lipoic acid attenuates renal injury in rats with obstructive nephropathy. Biomed Res Int.

[CR58] Xu T, Liu R, Zhu H (2022). The inhibition of LPS-induced oxidative stress and inflammatory responses is associated with the protective effect of (-)-epigallocatechin-3-gallate on bovine hepatocytes and murine liver. Antioxidants.

[CR59] Ying Z, Kampfrath T, Sun Q (2011). Evidence that α-lipoic acid inhibits NF-κB activation independent of its antioxidant function. Inflamm Res.

[CR60] Yoo JY, Cha DR, Kim B (2020). LPS-induced acute kidney injury is mediated by Nox4-SH3YL1. Cell Rep.

[CR61] Zhang J, McCullough PA (2016). Lipoic acid in the prevention of acute kidney injury. Nephron.

[CR62] Zhang P, Li T, Wu X (2020). Oxidative stress and diabetes: antioxidative strategies. Front Med.

[CR63] Ziegler D, Porta M, Papanas N (2022). The role of biofactors in diabetic microvascular complications. Curr Diabetes Rev.

